# Mutualistic Polydnaviruses Share Essential Replication Gene Functions with Pathogenic Ancestors

**DOI:** 10.1371/journal.ppat.1003348

**Published:** 2013-05-09

**Authors:** Gaelen R. Burke, Sarah A. Thomas, Jai H. Eum, Michael R. Strand

**Affiliations:** Department of Entomology, University of Georgia, Athens, Georgia, United States of America; Stanford University, United States of America

## Abstract

Viruses are usually thought to form parasitic associations with hosts, but all members of the family *Polydnaviridae* are obligate mutualists of insects called parasitoid wasps. Phylogenetic data founded on sequence comparisons of viral genes indicate that polydnaviruses in the genus *Bracovirus* (BV) are closely related to pathogenic nudiviruses and baculoviruses. However, pronounced differences in the biology of BVs and baculoviruses together with high divergence of many shared genes make it unclear whether BV homologs still retain baculovirus-like functions. Here we report that virions from *Microplitis demolitor* bracovirus (MdBV) contain multiple baculovirus-like and nudivirus-like conserved gene products. We further show that RNA interference effectively and specifically knocks down MdBV gene expression. Coupling RNAi knockdown methods with functional assays, we examined the activity of six genes in the MdBV conserved gene set that are known to have essential roles in transcription (*lef-4, lef-9*), capsid assembly (*vp39, vlf-1*), and envelope formation (*p74, pif-1*) during baculovirus replication. Our results indicated that MdBV produces a baculovirus-like RNA polymerase that transcribes virus structural genes. Our results also supported a conserved role for *vp39, vlf-1, p74*, and *pif-1* as structural components of MdBV virions. Additional experiments suggested that *vlf-1* together with the nudivirus-like gene *int-1* also have novel functions in regulating excision of MdBV proviral DNAs for packaging into virions. Overall, these data provide the first experimental insights into the function of BV genes in virion formation.

## Author Summary

Microorganisms form symbiotic associations with animals and plants that range from parasitic (pathogens) to beneficial (mutualists). Although numerous examples of obligate, mutualistic bacteria, fungi, and protozoans exist, viruses are almost always considered to be pathogens. An exception is the family *Polydnaviridae*, which consists of large DNA viruses that are obligate mutualists of insects called parasitoid wasps. Prior studies show that polydnaviruses in the genus *Bracovirus* evolved approximately 100 million years ago from a group of viruses called nudiviruses, which are themselves closely related to a large family of insect pathogens called baculoviruses. Polydnaviruses are thus of fundamental interest for understanding the processes by which viruses can evolve into mutualists. In this study we characterized the composition of virus particles from *Microplitis demolitor* bracovirus (MdBV) and conducted functional experiments to assess whether BV genes share similar functions with related essential baculovirus replication genes. Our results indicate that several genes in MdBV retain ancestral functions, but select other genes have novel functions unknown from baculoviruses. Our results also provide the first experimental data on the function of polydnavirus replication genes and enhance understanding of the similarities between these viruses and their pathogenic ancestors.

## Introduction

Microorganisms form associations with metazoan hosts that range from beneficial symbiosis (mutualists) to parasitic (pathogens). Mutualists serve as important sources of evolutionary innovation for hosts, while pathogens often acquire genes from hosts or other organisms that facilitate their own survival and cause disease. Although most research on obligate mutualists focuses on bacteria, several fungi and protozoans are also known to form beneficial partnerships [1]–[3]. Viruses in contrast are almost always thought to form parasitic associations [4]–[6]. A notable exception to this is the family *Polydnaviridae*, which consists entirely of large DNA viruses that are obligate mutualists of insects called parasitoid wasps [7], [8]. Polydnaviruses (PDVs) thus provide an opportunity for understanding the adaptations involved in the evolution of viruses into mutualists from pathogenic ancestors.

Parasitoid wasps reproduce by laying eggs into other insects (hosts) that their progeny consume [9]. The *Polydnaviridae* consists of two genera: the *Bracovirus* (BV) associated with ca. 20,000 species of wasps in the family Braconidae, and the *Ichnovirus* (IV) associated with ca. 18,000 species of wasps in the family Ichneumonidae [10]. Each wasp species carries a genetically unique PDV that persists in all cells as an integrated provirus. Viral replication only occurs in pupal and adult stage female wasps in a type of cell in the ovary called calyx cells. Virions from calyx cells are released via cell lysis and accumulate to high density in the lumen of the reproductive tract to form calyx fluid. Virions are also enveloped and contain multiple, circular, double-stranded DNAs of large aggregate size (190–600 kbp) that encode many virulence genes. Most PDV-carrying wasps parasitize larval stage Lepidoptera (moths) by depositing eggs containing the proviral genome plus a quantity of virions. These virions rapidly infect host cells, which is followed by expression of virulence genes that immunosuppress and alter the development of hosts in ways that are essential for survival of the wasp's progeny [11].

The origin and genomic organization of IVs is unclear [11]. In contrast, BV genomes exhibit features unlike any other known viruses [12]–[14]. As proviruses, their genomes consist of two types of DNA domains: those that contain genes with predicted roles in replication, and others that contain the virulence genes that become packaged into virions. Remarkably, these domains reside in different locations in the wasp genome [12], [13]. In addition, while genes with predicted roles in replication are transcribed in calyx cells, their transmission is entirely vertical and independent of any viral DNA replication or encapsidation [7], [11], [12]. Virulence gene-containing domains are likewise transmitted vertically. However, they also are amplified, excised from the wasp genome into circular forms and packaged into virions during replication in calyx cells [7], [15]–[20]. In all other cells of the wasp including the germ line both the replication and virulence genes of the proviral genome are silent [11]. BVs cause no apparent disease in wasps because almost no virulence genes are expressed in wasp cells and lytic replication is restricted to only calyx cells [11], [21]. In contrast, BVs cause severe disease in the hosts wasps parasitize because virions systemically infect the host insect and all of the virulence genes virions deliver are expressed [11], [21], [22]. The disease symptoms BVs cause in the host, however, are also essential for development of the wasp's offspring. Thus, BVs depend on wasps for genetic transmission, while wasps depend on BVs for parasitism of hosts.

**Figure 1:**
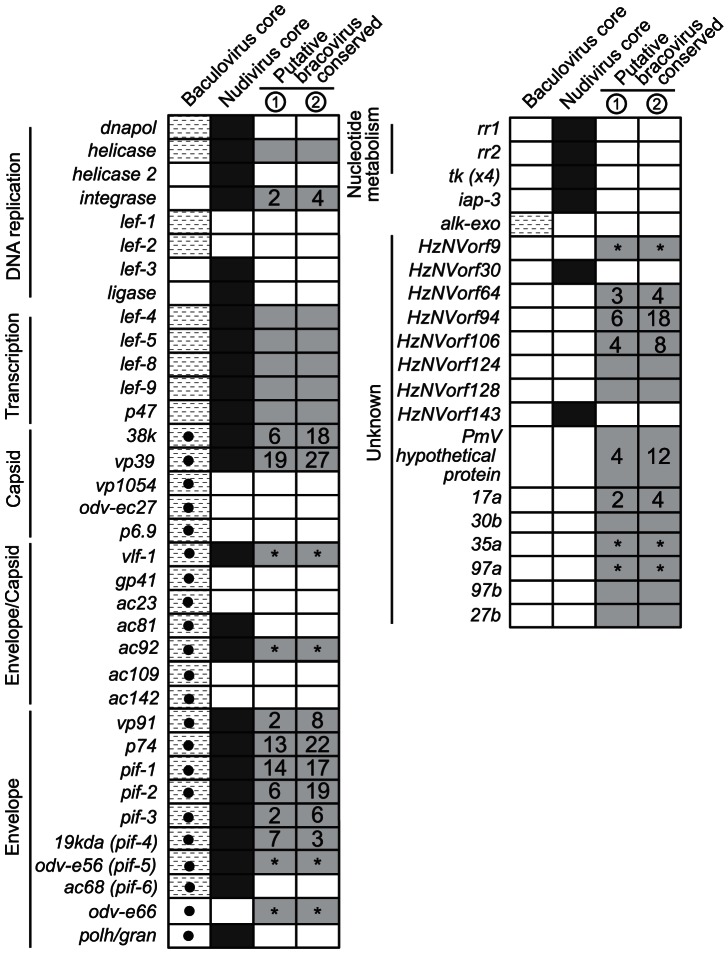
BV conserved gene products detected in MdBV virions. The predicted conserved gene set for BVs (gray boxes) is shown to the right relative to the predicted core genes for nudiviruses (black boxes) and baculoviruses (hatched boxes) as previously detailed [7]. Black circles in hatched boxes represent baculovirus core gene products that are detected in mature occlusion-derived baculovirus virions [55]. Two independently prepared protein samples for MdBV virions were analyzed and are indicated as replicate 1 and 2 in the figure. Each gray box for the two replicates with a number or asterisk (*) indicates that peptides matching this conserved gene product were detected. The number in each gray box indicates the total number of unique peptides that matched the product. Gray boxes with asterisks indicate that the MdBV proviral genome contains two or more homologs of nudivirus/baculovirus genes and peptides matching multiple homologs were identified (see Table S1). Gray boxes with no number or asterisk indicate that no peptides were identified that matched the product.

Genes in the proviral genome of BVs with predicted roles in replication were identified because they exhibit homology with core genes from two other types of arthropod-infecting viruses: nudiviruses and their sister taxon the *Baculoviridae* [7], [12]. Like BVs, nudiviruses and baculoviruses replicate in cell nuclei and package large, circular ds-DNA genomes into enveloped virions. Unlike the developmentally-linked and tissue-specific replication of BVs, however, baculoviruses are virulent pathogens, which establish systemic infections in insects by undergoing lytic replication in virtually all cells of the infected host and expressing a variety of virulence genes [23]. Nudiviruses produce either systemic, lytic infections or latent infections [23], [24]. More than 60 baculoviruses have been sequenced and a survey of a subset (13) of these genomes indicates that all share 31 genes, which are collectively referred to as the baculovirus core gene set [24], [25] (Figure 1). Functional studies of model species like *Autographa californica* multinucleopolyhedrosis virus (AcMNPV) indicate about half of these genes have essential roles in viral replication [24]. These include genes with roles in replicating the viral genome, several genes that code for virion structural components, and four genes that code for subunits of a novel RNA polymerase, which selectively transcribes viral genes because it recognizes unique promoter sequences (Figure 1) [24]. Six nudivirus genomes have been sequenced and each contains 20 genes with homology to structural, replication and transcription components of the baculovirus core gene set [23] (Figure 1). The actual function of these genes, however, is unknown beyond inferences from baculoviruses. Data from three braconid wasps, *Cotesia congregata, Chelonus inanitus*, and *Microplitis demolitor*, indicate they lack recognizable homologs of most baculovirus core genes with roles in viral DNA replication (Figure 1), which suggests that, unlike baculoviruses, replication of BV DNAs packaged into virions is regulated by machinery from the wasp [7]. However, BVs do encode homologs of several baculovirus/nudivirus-like structural genes plus the four subunits of a baculovirus/nudivirus-like RNA polymerase [7], [12]. Each of these genes is also transcribed in ovaries when BV virions are produced. Together, these genes form a conserved gene set likely present in all BV genomes (Figure 1). However, we refrain from referring to these as “core” genes because of the small number of BV genomes currently available and their non-discrete organization in wasp genomes [7]. Other predicted members of a conserved BV gene set include a baculovirus/nudivirus-like sulfhydryl oxidase (*ac92*), 11 nudivirus-like genes unknown from baculoviruses, and 11 novel genes [7].

Since BV-carrying braconids are monophyletic [26], these data overall indicate that BVs evolved from an ancestral nudivirus-wasp association. Fossil calibrations estimate this association arose 100 million years ago (Mya), while the last common ancestor of BVs, nudiviruses, and baculoviruses existed approximately 312 Mya [27]. Given these timelines and the pronounced differences that exist today between BVs and baculoviruses, it is not surprising many of the genes they share have diverged to the point that homology is difficult to recognize outside of essential residues or functional domains. Indeed, algorithms like BLAST cannot detect homology between BV and baculovirus genes, while identity between predicted BV and more closely related nudivirus proteins ranges between 19–41% [7]. Such divergence, however, also begs the question of whether baculovirus-like genes in BV proviral genomes retain baculovirus-like functions. Here, we used proteomic, RNA interference (RNAi), and functional assays to characterize selected members of the conserved gene set of *Microplitis demolitor* bracovirus (MdBV) in the wasp *M. demolitor*. Our results indicate that six genes with hypothesized roles in replication exhibited conserved functions relative to baculoviruses. Our data also identified novel functions for two genes in excision of viral DNAs for packaging into virions.

## Results

### MdBV virions contain conserved viral gene products

BV replication in calyx cells begins with amplification of a portion of the proviral genome, which is followed by the *de novo* assembly and packaging of virions in nuclei [20], [28]. Calyx cells then lyse which releases virions into the lumen. In the case of MdBV, prior studies establish the timing of these events and the chronology of replication gene expression during the pupal and adult stages of *M. demolitor* [7]. MdBV packages multiple circular, double-stranded DNA segments into virions but each individual virion contains only a single viral DNA [14], [29]. The sequence of these DNAs as episomes and their wasp-viral boundary sequences when integrated into the genome of *M. demolitor* are known [14], [29], [30]. Prior studies document that these DNAs are specifically amplified in *M. demolitor* calyx cells, followed by excision from flanking DNA and circularization [7]. Flanking wasp DNA at the site of excision is then rejoined to form an ‘empty locus’ [7].

Given this background, we first conducted a proteomic analysis of MdBV virions to determine whether predicted conserved structural components were present. To accomplish this, we produced two independent samples of calyx fluid with the second sample further purified on a sucrose gradient that produces morphologically pure and intact virions [31]. Following separation on SDS-PAGE gels, proteins were in-gel trypsin digested and analyzed using an Orbitrap Elite mass spectrometer. Mass spectra were then compared to our previously generated *M. demolitor* ovary transcriptome database [7] to identify proteins present. We present our findings relative to the predicted core/conserved gene sets for baculoviruses, nudiviruses, and BVs in Figure 1 and Table S1. Four proteins (38K, VP39, VLF-1, AC92) detected in MdBV virions were homologs of baculovirus capsid or capsid/envelope components. VP39 was the most accurately detected of these proteins based on the total number of unique peptides identified, which corresponded with *vp39* also being the most abundant viral gene transcript detected in ovaries during MdBV replication [7] (Table S1). We also detected several proteins related to envelope components of baculovirus occlusion-derived virus. These included variants of ODV-E66 and ODV-E56 ( = PIF-5) plus the infectivity factors P74, PIF-1 through -6 and envelope component VP91 (Figure 1, Table S1). Seven MdBV virion proteins corresponded to genes in the BV conserved gene set for which homologs are known from all or some nudiviruses but are unknown from baculoviruses (Figure 1). These included the product of the *integrase-1* (*int-1*) gene, which is structurally related to *vlf-1*, plus products of several nudivirus-like genes of unknown function (HzNVORF9-1 and -2, 64, 94, 106, PMV Hypothetical Protein). We also detected products of four conserved genes or gene families unique to BVs (17A, 35A, 97A) (Figure 1, Table S1). In contrast, we did not detect any proteins in virions that corresponded to the *helicase* gene or the RNA polymerase subunits (*lef-4, lef-8, lef-9, p47*) (Figure 1).

### MdBV conserved genes are efficiently knocked down by RNAi

The preceding data showed that MdBV virions contain BV conserved gene products but provided no experimental evidence for their function. We therefore selected six genes from MdBV for functional studies. Our choices included two predicted subunits of a baculovirus-like RNA polymerase (*lef-4, lef-9*), 2), two homologs of baculovirus capsid genes (*vp39*, and *vlf-1*), and 3) two homologs of baculovirus envelope genes (*p74* and *pif-1*). Each of these genes is a member of the BV conserved gene set because orthologs are likely present in all other BVs studied to date (see Figure 1). Each is also a member of the baculovirus core gene set with prior studies from AcMNPV or other isolates providing experimental evidence for the function of each [24]. In addition, we selected one nudivirus-like gene (*int-1*), unknown from baculoviruses, for which no functional studies have been conducted. As previously noted, sequence divergence between members of the BV conserved gene set and corresponding baculovirus/nudivirus core genes is high. Identities of the above gene products from MdBV with corresponding predicted proteins from the closest known nudivirus relative, *Heliothis zea* nudivirus-1 (HzNV-1), were: *lef-4* (25%), *lef-9* (32%), *vp39* (19%), *vlf-1* (28%), *p74* (26%), *pif-1* (28%), and *int-1* (30%).

**Figure 2:**
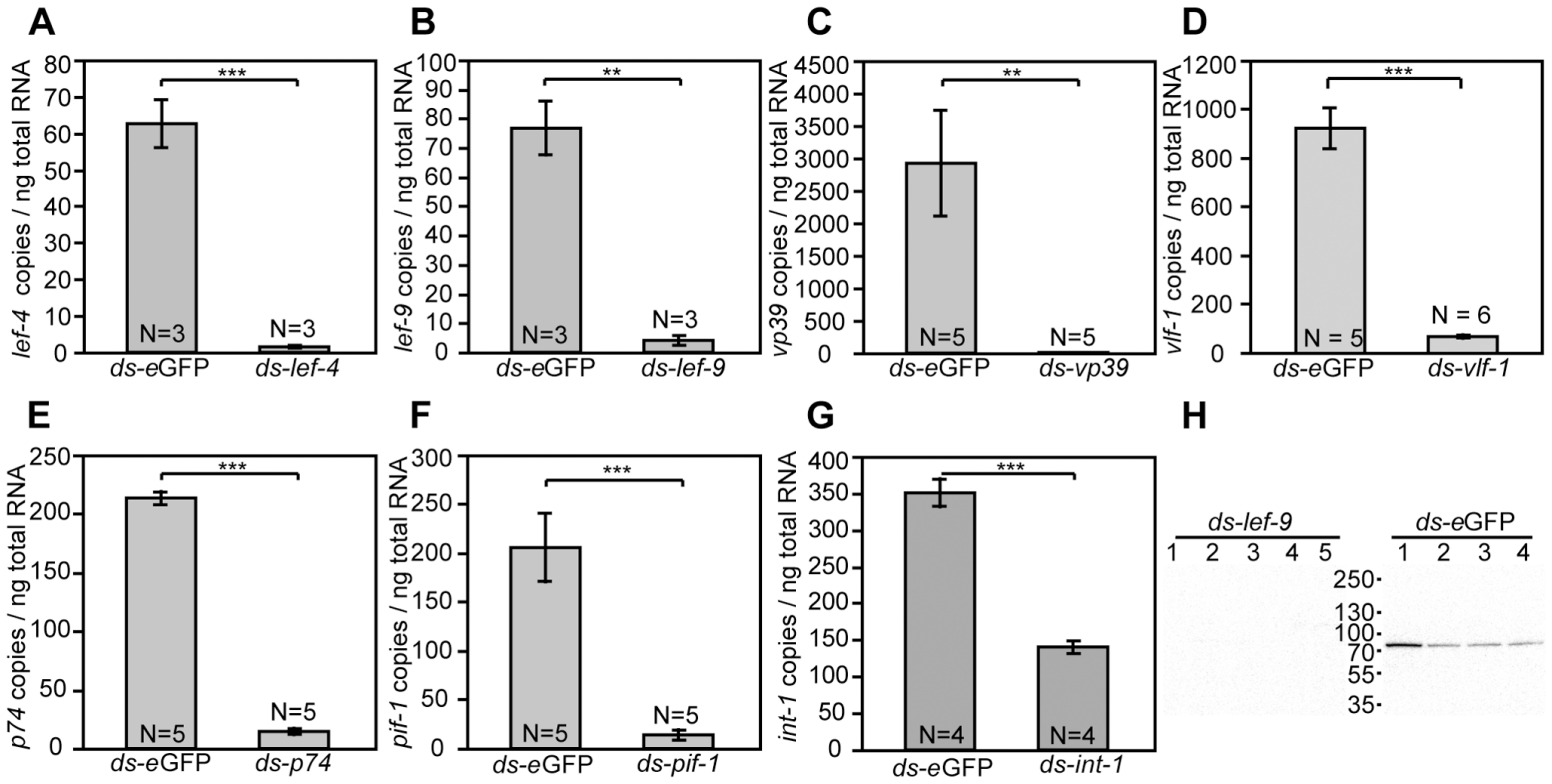
RNAi knockdown of six MdBV putative replication genes. *M. demolitor* larvae were injected with double-stranded RNA (dsRNA) specific for (A) *lef-4*, (B) *lef-9*, (C) *vp39*, (D) *vlf-1*, (E) *p74*, (F) *pif-1*, (G) *int-1*. Control larvae were injected with ds-*eGFP*. The ovaries from individual, newly emerged adults wasps were then dissected and total RNA isolated. The bars in each graph compare copy number of each target gene per ng of total RNA in wasps treated with dsRNA specific for the target gene versus ds-*eGFP* (control). Error bars represent one standard error from the mean. The number of individuals examined for each treatment is indicated by the N value at the bottom of each bar. Statistical significance is indicated by asterisks: *, *p*<0.05; **, *p*<0.001; ***, *p*<0.0001; N. S., not significant. (H) Knockdown of *lef-9* depletes LEF-9 protein. *M. demolitor* larvae were injected with ds-*lef-9* as in (B). Total protein from the ovaries of a newly emerged adult wasp was loaded into lanes of an SDS-PAGE gel followed by immunoblot analysis using an MdBV anti-LEF-9 antibody (1∶5000). The left lanes show results from 5 individual *M. demolitor* females treated with ds-*lef-9* (knockdown), while the lanes on the right show results from 4 females treated with ds-*eGFP* (control). The predicted size of MdBV LEF-9 is 74.4 kDa.

The genes we selected reside in the MdBV proviral genome and each is transcribed in ovary calyx cells during replication [7]. However, conventional knock out techniques used to characterize baculovirus gene function are untenable because the DNA domains where these genes reside are not replicated and packaged into MdBV virions. We thus assessed whether RNAi could be used to knock down transcription of these genes in *M. demolitor*. Since MdBV replication begins in the pupal stage of the wasp, we developed methods for injecting gene-specific dsRNAs into wasp larvae after they emerged from a host caterpillar and spun a cocoon. We then compared the abundance of each targeted transcript in newly emerged adult wasps by qPCR relative to treatment with a non-specific dsRNA (ds-*eGFP*). Our results showed that we reduced transcript abundance on average 60–99% for each gene we targeted (Figure 2). Using an antibody we generated to MdBV LEF-9, we also confirmed that knockdown at the transcript level resulted in knockdown of the corresponding protein (Figure 2).

Before initiating any functional experiments, we further verified our approach by examining the effects of dsRNA dose, time required after treatment for target transcript degradation, and specificity. Using *vlf-1* as an example, our results showed that injection of 50 ng to 5 µg of dsRNA per wasp larva yielded a similar level of knockdown (Figure S1A). Our results also indicated that injection of *vlf-1* dsRNA into wasp larvae did not significantly reduce transcript abundance until day 3 of the pupal stage, which suggested that 2–3 days were required before an RNAi effect was observed (Figure S1B). We examined specificity of knockdown in two ways. Since *vlf-1* and *int-1* are homologous genes, we verified that *int-1* dsRNA, which strongly knocked down *int-1* (Fig. 2G), did not affect transcript abundance of *vlf-1* via off-target effects [32] (Figure S2A). We also generated a second *vlf-1* dsRNA that did not overlap the dsRNA used for the data presented in Figure 2D to verify that it had a similar knockdown effect on *vlf-1* transcript abundance (Figure S2B).

### MdBV LEF-4 and LEF-9 are RNA polymerase subunits

Baculovirus RNA polymerases consist of four subunits (LEF-4, LEF-8, LEF-9, P47), which transcribe baculovirus genes with roles in virion formation [33]. These subunits are categorized as ‘early’ genes because they are transcribed before DNA replication and transcription of structural ‘late’ and ‘very late’ genes commences. As noted above, baculovirus RNA polymerases selectively transcribe late and very late viral genes because they recognize unique promoter elements with the consensus sequence (A/G/T)TAAG absent from host insect genes [34], [35]. The conserved gene set of BVs contains homologs of each RNA polymerase subunit. Expression data from *M. demolitor* and *Cotesia congregata* also indicate these subunits are transcribed in ovaries earlier than predicted structural genes, while sequence analysis has identified baculovirus late gene promoter elements upstream of the start codon of several predicted BV structural genes [7], [12]. Thus, if the BV RNA polymerase subunits form a functionally similar enzyme as baculoviruses, RNAi knockdown of one or more subunits should compromise transcription of MdBV structural genes but not wasp genes.

**Figure 3:**
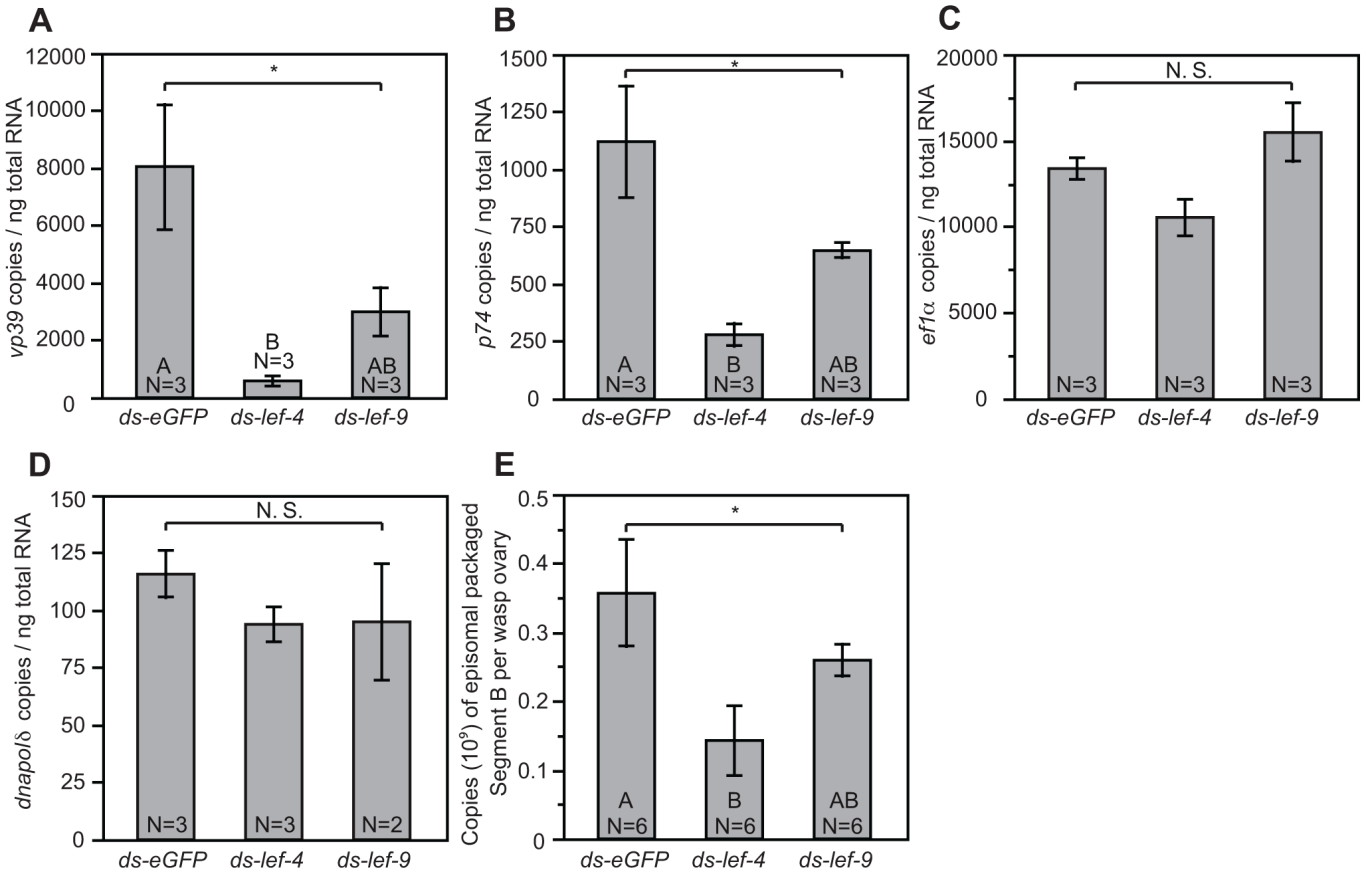
Knockdown of *lef-4* and *lef-9* reduces expression of MdBV structural genes. *M. demolitor* larvae were injected with ds-*eGFP*, ds-*lef-4* or ds-*lef-9* as shown in Figure 2. The ovaries from individual, newly emerged adults wasps were then dissected and total RNA isolated followed by measurement of copy number per ng of total RNA of: (A) *vp39*, (B) *p74*, (C) *ef1α*, (D) *dnapolδ*. (E) Copy number of DNase-protected MdBV episomal genomic segment B in the ovaries of newly emerged *M. demolitor* adult females. The ovaries from individual, newly emerged adults wasps were dissected and treated with DNAse. DNA was then isolated and total copy number of episomal genomic segment B determined. Error bars, N values, and statistical significance are indicated as defined in Figure 2.

As shown above (Figure 2A, B), we knocked down *lef-4*, a predicted 5′ capping enzyme [36], [37] and *lef-9*, a predicted RNA polymerase subunit that forms part of the catalytic cleft [38], [39]. We then measured transcript abundance of two predicted MdBV structural genes (*vp39* and *p74*), and two typical insect genes expressed in ovaries (elongation factor 1 alpha (*ef1α*) and DNA polymerase delta subunit (*dnapolδ*)). Our results showed that knockdown of *lef-4* and *lef-9* significantly reduced transcript abundance of *vp39* and *p74* while having no effect on *ef1α* and *dnapolδ* (Figure 3A–D). Given this outcome and the putative role of *vp39* and *p74* as structural genes we reasoned that reduced expression of *vp39* and *p74* could also result in production of fewer virions on average than control females. We therefore estimated viral titer by using episomal MdBV DNA segment B as a marker and a previously developed qPCR assay that includes a DNAse step to remove all non-encapsidated DNA before isolating DNA from ovary homogenates [7]. In this manner, the copy number of episomal segment B in virions, which protect the packaged DNA, could be determined. These results showed that knockdown of *lef-4* and *lef-9* significantly reduced the titer of DNAse-protected segment B relative to ds-eGFP-treated controls (Figure 3E).

### VP39 and VLF-1 likely encode structural components of viral nucleocapsids

**Figure 4:**
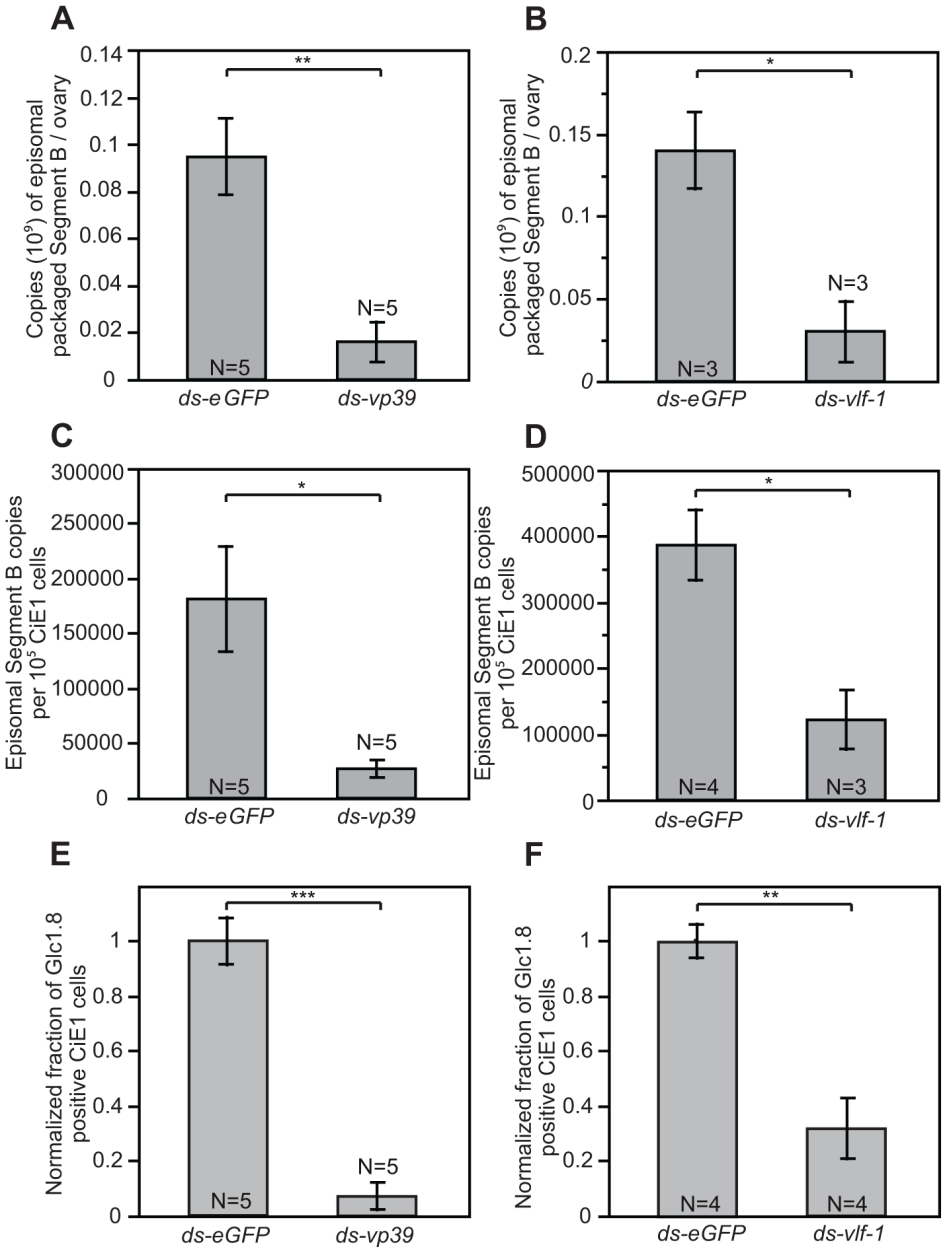
Knockdown of *vp39* and *vlf-1* increases DNAse sensitivity and reduces infectivity. *M. demolitor* larvae were injected with ds-*eGFP*, ds-*vp39* or ds-*vlf-1* as shown in Figure 2. (A) Copy number of DNase-protected MdBV episomal genomic segment B in the ovaries of newly emerged *M. demolitor* adult females pretreated with ds-*eGFP* or ds-*vp39*. (B) Copy number of DNase-protected MdBV episomal genomic segment B in the ovaries of newly emerged *M. demolitor* adult females pretreated with ds-*eGFP* or ds-*vlf-1*. (C) Copy number of MdBV episomal genomic segment B in CiE1 cells infected with MdBV from wasps pretreated with ds-*eGFP* or ds-*vp39*. (D) Copy number of MdBV episomal genomic segment B in CiE1 cells infected with MdBV from wasps pretreated with ds-*eGFP* or ds-*vlf-1*. (E) Normalized fraction of CiE1 cells expressing the gene product GLC1.8 on their surface after infection with MdBV from wasps pretreated with ds-*eGFP* or ds-*vp39*. (F) Normalized fraction of CiE1 cells expressing the gene product GLC1.8 on their surface after infection with MdBV from wasps pretreated with ds-*eGFP* or ds-*vlf-1*. Error bars, N values, and statistical significance are indicated as defined in Figure 2.

In baculoviruses, VP39 is a major capsid protein while VLF-1 is a structural component, and is also functionally required for capsid production and very late gene expression [24], [40]–[42]. After knocking down *vp39* and *vlf-1* in *M. demolitor* (Figure 2C, D), we first assessed whether either treatment affected virion structural integrity by measuring the DNase sensitivity of packaged genomic DNAs as described above. These assays indicated that the abundance of DNase-protected segment B declined by 83% and 78% in *vp39* and *vlf-1* knockdown samples respectively relative to the control (Figure 4A, B). We also used the non-overlapping dsRNA, ds-*vlf-1-2* in these assays, which produced the same result as ds-*vlf-1* (Figure S2C).

We then examined the effects of *vp39* and *vlf-1* knockdown on the ability of MdBV to infect cells from the moth *Chrysodeixis includens*, which is a host for *M. demolitor*. For these assays, we used CiE1 cells, which is a continuous, hemocyte-like cell line established from *C. includens* that is highly permissive to MdBV infection [30], [43]. We determined by qPCR that 2–4 copies of episomal DNA segment B were present per CiE1 cell when cultures were infected at an estimated MOI of 100 with MdBV from control wasps treated with ds-eGFP (Figure 4C, D). In contrast, copy number was 85.2% and 69.6% less when cells were infected with the same amount of calyx fluid from *vp39* and *vlf-1* knockdown wasps. We also assessed infection using the MdBV gene product GLC1.8, which is an excellent marker because it is rapidly expressed on the surface of CiE1 cells and is easily visualized immunocytochemically [43], [44]. Normalizing the control samples, we observed that *vp39* knockdown reduced the fraction of cells stained for GLC1.8 to less than 10%, while *vlf-1* knockdown reduced this fraction to 26.8% (Figure 4E,F).

**Figure 5:**
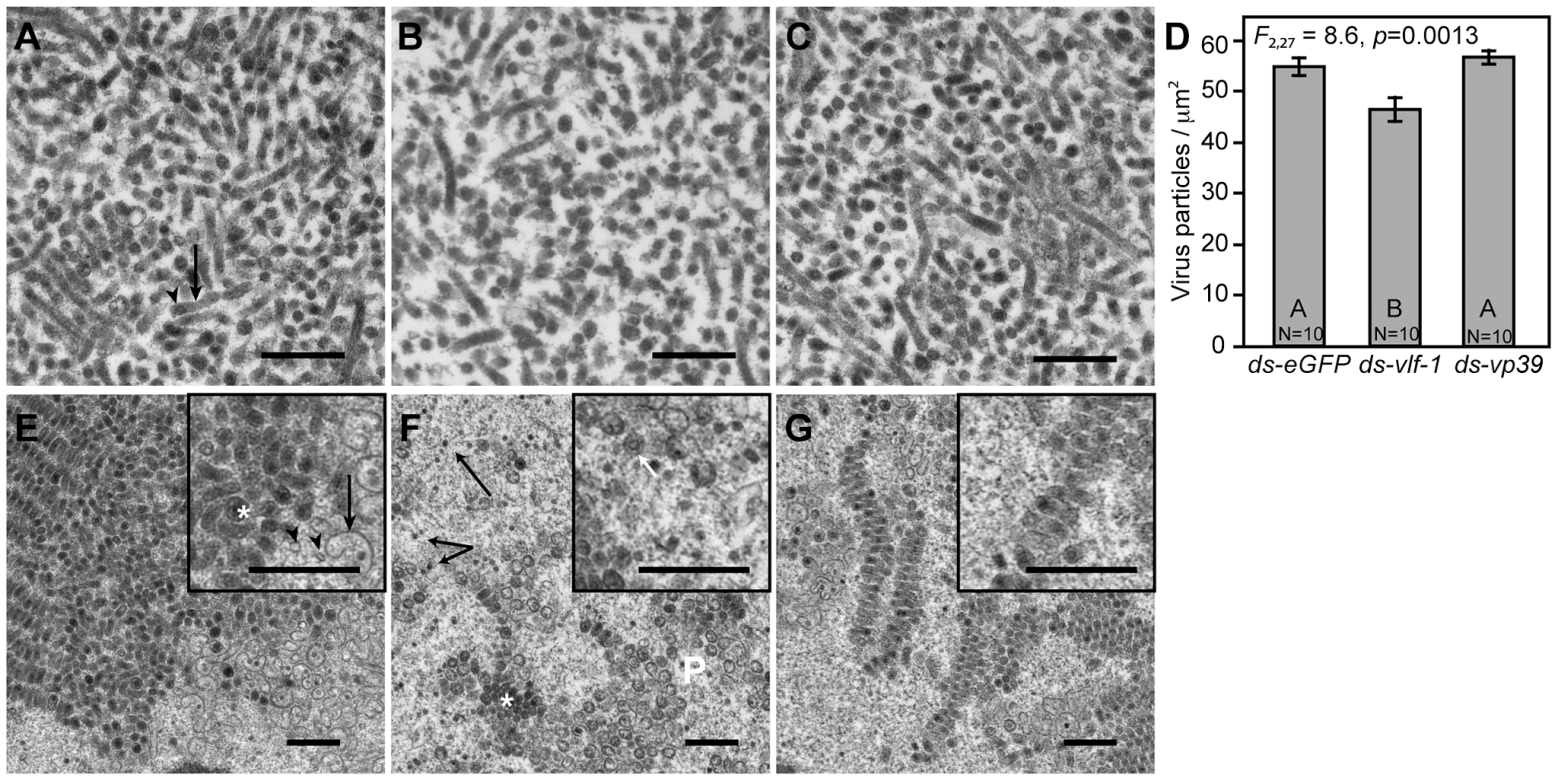
Electron microscopy analysis shows defects in MdBV morphogenesis after *vlf-1* and *vp39* knockdown. *M. demolitor* larvae were injected with ds-*eGFP*, ds-*vp39* or ds-*vlf-1* as shown in Figure 2. (A) Image of calyx fluid in the lumen of the reproductive tract of a newly emerged adult female pretreated with ds-*eGFP*. Each virion consists of one electron dense nucleocapsid (arrowhead) surrounded by a single elongate envelope (arrow). (B) Image of calyx fluid from a wasp pretreated with ds-*vlf-1*. Note the lower density of virions present relative to (A). (C) Image of calyx fluid from a wasp pretreated with ds-*vp39*. Scale bars in A-C equal 500 nm. (D) Graph showing that virion density in calyx fluid from wasps treated with ds-*vlf-1* was significantly lower than in wasps treated with ds-*eGFP* or ds-*vp39*. (E) Image showing a portion of a calyx cell nucleus from a wasp pretreated with ds-*eGFP*. The left side of the image shows a large array of assembled MdBV virions. Virions in the process of assembly are visible in the lower right of the image. The insert in the upper right shows virion assembly at higher magnification. Note that assembled virions (*), *de novo* forming envelopes, empty capsids (arrowhead) and electron dense nucleocapsids in the process of being surrounded by envelopes (arrow). (F) Image showing a portion of a calyx cell nucleus from a wasp pretreated with ds-*vlf-1*. Unlike normal calyx cells, few virions are assembled into arrays (*). Most of these virions have envelopes that are rounded. Numerous electron dense nucleocapsids without envelopes (arrows) and rounded envelopes without electron dense nucleocapsids are visible. The insert in the upper right shows these defects at higher magnification. It also shows that some rounded envelopes contain empty capsids (arrow). (G) Image showing a portion of a calyx cell nucleus from a wasp pretreated with ds-*vp39*. Arrays of assembled virions are visible but they are much smaller than the arrays observed in calyx cell nuclei from control wasps. The insert in the upper right shows virions with elongate envelope and electron dense nucleocapsid similar to virions from control wasps. Scale bar for images and inset images E–G equal 500 nm.

These results could be explained by *vp39* and *vlf-1* knockdown either adversely affecting virion formation, which would result in calyx fluid containing a lower titer of virus, or causing structural defects that do not reduce virion density but nonetheless compromise function. We therefore examined virion morphology in calyx fluid by transmission electron microscopy (TEM). We previously documented that MdBV virions in calyx fluid consist of a single barrel-shaped nucleocapsid surrounded by a highly elongate envelope [29], [31]. By counting the number of virions in randomly selected fields of view from treatment and control wasp sections, we determined that calyx fluid from a *vlf-1* knockdown wasp contained a slightly lower concentration of virions than observed in a control wasp, whereas a *vp39* knockdown wasp did not (Figure 5A–D).

We then examined MdBV morphogenesis in calyx cell nuclei. Early studies of BVs show that calyx cells exhibit a progression of development with smaller, younger cells being situated closer to the ovarioles and older, large cells being closer to the lumen of the ovary [45]. In turn, young cells show no evidence of BV replication, while old calyx cells contain an abundance of assembled virions in their nuclei [45], [46]. In control wasps, we observed that MdBV morphogenesis began with the *de novo* appearance of short membrane profiles in calyx cell nuclei. This was followed by the formation of nucleocapsids near virogenic stroma, which is where DNA packaging also occurs in baculoviruses. Assembled MdBV virions then formed large aggregations with a layered crystalline structure (Figure 5E). At this stage, virions were rod-shaped and of uniform length. The envelope surrounding each nucleocapsid was also not as elongated as seen for virions in calyx fluid (see Figure 5A versus 5E). Calyx cells from *vlf-1* knockdown wasps in contrast exhibited an abundance of membrane profiles that appeared to be envelope progenitors (Figure 5F). Rather than elongating, these envelopes were spherical and either lacked capsids entirely or contained empty capsids (Figure 5F). A number of electron dense and empty capsids were also observed with no envelope (Figure 5F). Lastly, almost no aggregations of rod-shaped, electron dense virions were present in calyx cells from *vlf-1* knockdown wasps. Calyx cells from *vp39* knockdown wasps showed no distinct alterations in virion assembly, but aggregations of rod shaped, electron dense virions were consistently much smaller than those observed in control wasps (Figure 5G).

### Knockdown of *p74* and *pif-1* reduces the fraction of host cells that express the marker GLC1.8

**Figure 6:**
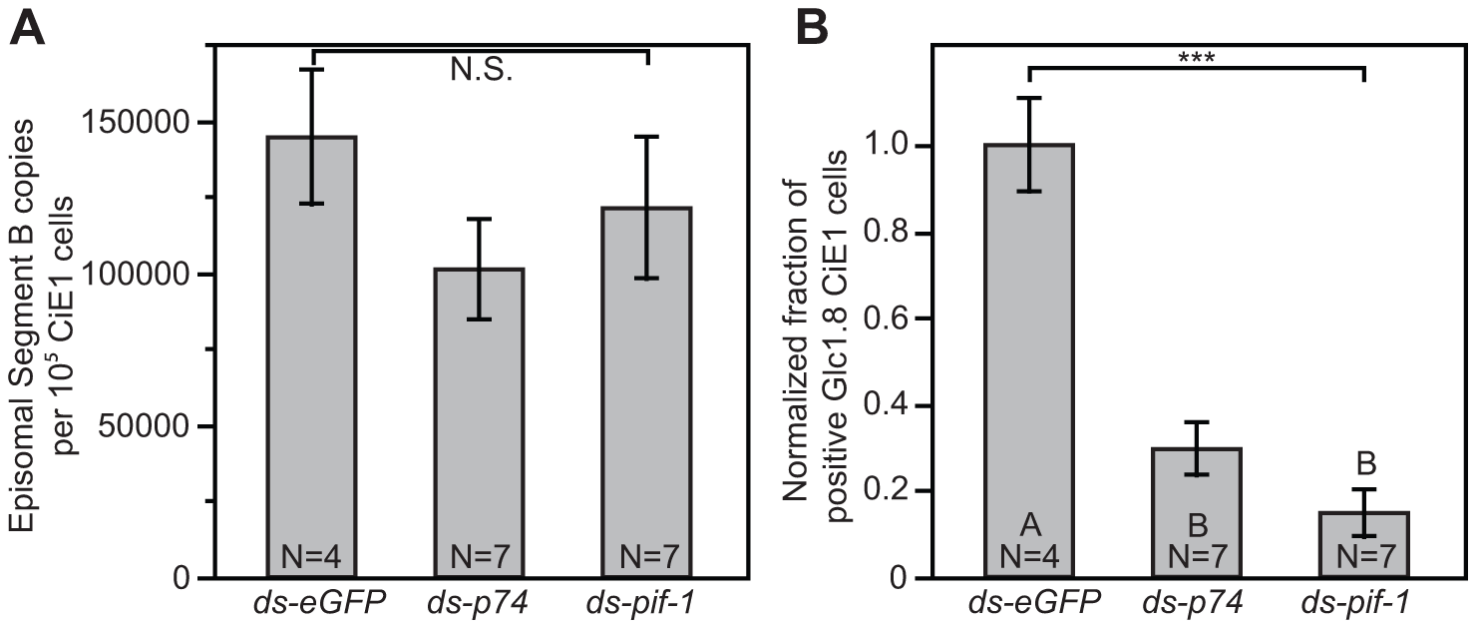
Knockdown of *p74* and *pif-1* reduces the fraction of host cells that express GLC1.8. *M. demolitor* larvae were injected with ds-*eGFP*, ds-*p74* or ds-*pif-1* as shown in Figure 2. (A) Copy number of MdBV episomal genomic segment B in CiE1 cells infected with MdBV from wasps pretreated with ds-*eGFP*, ds-*p74*, or ds-*pif-1*. (B) Normalized fraction of CiE1 cells expressing the gene product GLC1.8 on their surface after infection with MdBV from wasps pretreated with ds-*eGFP*, ds-*p74*, or ds-*pif-1*. Error bars, N values, and statistical significance are indicated as defined in Figure 2.

*p74* and the *pif* genes are known as *per os* infectivity factors because their loss in baculoviruses such as AcMNPV disables oral infection of host insects by occlusion derived virus [47]–[49]. Each is also a component of the occlusion derived virus envelope where they form a complex with one another [50]. Unlike baculoviruses, BV virions never infect host insects orally but instead are injected into the hemocoel by wasps where they bind to host cells such as hemocytes via fusion of the envelope with the plasma membrane [51]. Nucleocapsids then travel through the cytoplasm to nuclear pores where they release their DNA into the nucleus to initiate transcription of virulence genes like *glc1.8* [51], [52]. Given the differences in the known functions of *per os* infectivity factors in baculoviruses relative to the biology of BVs we asked whether the *p74* and *pif-1*-like genes of MdBV still play a role in infectivity by knocking down each (Figure 2E, F) and then conducting the same assays in CiE1 cells as described above. Our results revealed no differences between knockdown and control wasps in the copy number of DNA segment B in CiE1 cells at 24 h post-infection (Figure 6A). However, the fraction of CiE1 cells expressing GLC1.8 on their surface was dramatically lower using virus from *p74* and *pif-1* knockdown wasps (Figure 6B).

### Knockdown of VLF-1 and INT-1 disables proviral DNA excision

As noted above, all baculoviruses encode a *vlf-1* gene while nudiviruses and BVs also encode related *integrase* genes (known as *vlf-1* or *vlf-1a, vlf-1b-1* and *-2* or *HzNVORF140*, and *int-1* and *-2* or *HzNVORF144*) [7], [12]. Although VLF-1 is a structural component of baculovirus virions, it along with nudivirus integrase genes are members of the tyrosine (Tyr) recombinase family, which includes several enzymes that mediate the excision and integration of genetic elements [53]. As noted above, elimination of *vlf-1* from AcMNPV disables capsid formation while mutation of the conserved Tyr residue required for integrase activity in other Tyr recombinase family members produces non-infectious virus [40]. Whether baculovirus VLF-1 possesses any integrase activity, however, remains unstudied in all likelihood because baculovirus genomes persist as episomes in infected host cells and are unknown to integrate. In contrast, a key feature of BVs is their persistence in wasps as integrated proviruses that amplify, excise and package a portion of the genome when replicating in calyx cells. We therefore assessed whether *vlf-1* and/or *int-1* homologs from MdBV regulate proviral DNA excision.

**Figure 7:**
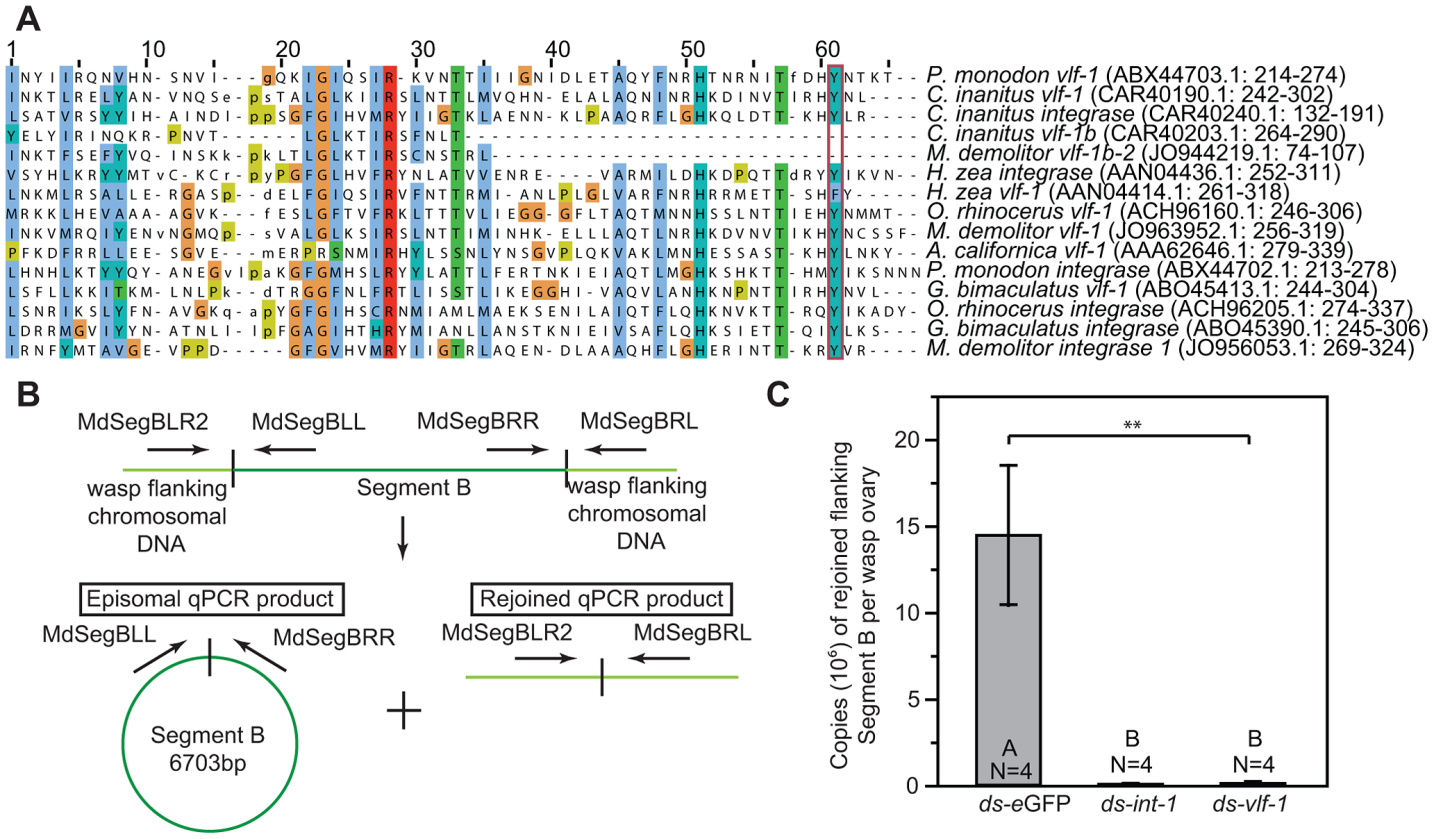
Knockdown of *vlf-1* and *int-1* disables excision of genomic segment B from the *M.* *demolitor* genome. (A) Alignment of MdBV *vlf-1* and *integrase* family members to family members from select nudiviruses (*Paenaeus monodon* nudivirus (PmNV), *Heliothis zea* nudivirus (HzNV-1), *Gryllus bimaculatus* nudivirus (GbNV), *Oryctes rhinoceros* nudivirus (OrNV)), the baculovirus *Autographa californica* multinnucleopolyhedrosis virus (AcMNPV), and *Chelonus inanitus* bracovirus (CiBV). The alignment shows only the portion of each where the predicted conserved tyrosine (Y) residue required for recombinase activity by other tyrosine recombinase family members is located (box). Select other conserved residues are highlighted with other background colors. (B) Schematic illustrating the qPCR assays to quantify copy number of MdBV genomic segment B (6307 bp) in *M. demolitor* ovaries [7], [14], [30]. Episomal segment B is specifically amplified using the primers MdSegBLL and MdSegBRR, which can only generate a product if segment B is circularized. Copy number of the rejoined empty locus in *M. demolitor* is specifically amplified using the primers MdSegBLR2 and MdSegBRL, which can only generate a product if segment B has been excised and the wasp flanking domains are rejoined. (C) Copy number of the rejoined empty locus from excision of segment B in the ovaries of newly emerged *M. demolitor* adult females pretreated with ds-*eGFP*, ds-*int-1* or ds-*vlf-1*. The ovaries from individual, newly emerged adults wasps were dissected, followed by DNA isolation and qPCR analysis as outlined in (B). Error bars, N values, and statistical significance are indicated as defined in Figure 2.

We had previously determined that the MdBV proviral genome encodes three distinct *vlf-1* genes (named *vlf-1, vlf1b-1, -2*) and two *integrase* genes (*int-1, -2*) that are all transcribed in ovaries during replication [7]. Phylogenetic analysis further suggested the *integrase* genes of BVs likely arose from duplication of *vlf-1* in the nudivirus ancestor, which was then followed by duplication of each gene in *M. demolitor* [7]. Alignment of *vlf-1* and *integrase* family members from MdBV, select nudiviruses, AcMNPV, and *Chelonus inanitus* bracovirus (CiBV) showed that MdBV *vlf-1* and *int-1* both retain a typical active site Tyr residue for predicted integrase activity, whereas other *M. demolitor* family members do not (Figure 7A). We thus knocked down *vlf-1* and *int-1* (Figure 2D, G), and then isolated DNA from newly emerged adult wasp ovaries to determine whether proviral DNAs had excised from the wasp genome as normally occurs. This was accomplished using MdBV segment B as a marker and qPCR assays that measured copy number of the rejoined ‘empty locus’ that only forms if proviral DNA segment B was excised from the wasp genome (Figure 7B). Copy number of the empty locus in ovaries from control wasps treated with ds-*eGFP* was 14.4×10^6^, which indicated a high level of excision of DNA segment B from calyx cells. In contrast, we detected almost no copies of the empty locus in wasps treated with ds-*int-1* and *ds-vlf-1* (Figure 7C).

## Discussion

Phylogenetic data strongly support that BVs evolved from a nudivirus ca. 100 Mya, and that nudiviruses and baculoviruses shared a more ancient common viral ancestor ca. 200 Mya earlier [12], [26], [27]. Detailed studies of AcMNPV and select other species also provide important insights into the function of baculovirus core genes. In contrast, the hypothesized function of baculovirus core gene homologs in BVs (and nudiviruses) is founded on inferences from the baculovirus literature and/or expression patterns in wasp ovaries during replication. Thus, the primary goal of this investigation was to assess whether RNAi methods could be used to disrupt BV gene expression, and then to use these methods with a subset of genes to determine whether their roles in replication were consistent with or differed from baculoviruses.

Prefacing these functional studies, we conducted a proteomic analysis of purified MdBV virions to assess whether BV conserved genes that are homologs of baculovirus structural components were present. We also did this to compare MdBV virions to virions from CcBV and CiBV, which are the only other BVs for which any proteomic data are available [12], [54]. We detected all of the baculovirus-like capsid and envelope components previously identified in CcBV and CiBV as well as two additional baculovirus-like conserved genes not detected in CcBV or CiBV. These included AC92, which is associated with baculovirus nucleocapsids, and PIF-3, which is associated with baculovirus occlusion-derived virus envelopes [55]. We also detected four nudivirus-like (HzNVORF9-1 and -2, HzNVORF106, PmV) and three novel conserved gene products (17A, 35A, 97A) in MdBV virions identified in CiBV virions. In contrast, we did not detect three novel gene products (27B, 30B, 97B) reported from CiBV, yet did detect three nudivirus-like gene products (INT-1, HZNVORF64, HZNVORF94) not reported from CiBV virions.

Proteomic data must be interpreted cautiously when investigating viral structural proteins, because of the potential for non-integral proteins to become non-specifically associated with virions during assembly [24]. Nonetheless, the baculovirus literature combined with our detection of the capsid and envelope proteins in Figure 1 suggest these baculovirus-like gene products are likely structural components of MdBV virions. While no functional data from nudiviruses exist, we speculate for the same reason that products of the nudivirus-like conserved genes *HzNVorf 9-1, 9-2, -64, -94, -106*, *PmV hypothetical protein* and novel conserved genes *17a, 35a*, and *97a*, are also structural proteins. Our proteomic data did not identify any peptides corresponding to MdBV conserved genes with predicted roles in DNA replication (*helicase*) or transcription (*lef-4, lef-8, lef-9, p47*), which at minimum indicates our samples were not contaminated with some non-integral products transcribed in calyx cells [7]. However, it is notable that we consistently detect the products of the *int-1, vlf-1b-1* and *vlf-1b-2* genes, which could suggest that similar to baculovirus *vlf-1* they too are capsid components or are packaged into capsids together with episomal DNA.

Because of the unique biology of BVs and limited genetic data available for their associated wasps, the options available for studying gene function are obviously constrained. RNAi is a potentially powerful method for studying BV gene function, but its efficacy in insects is also patchy with examples of successful use being more prevalent in some taxa (beetles (Coleoptera), mosquitoes (Diptera)) [56], [57] than others (moths (Lepidoptera) [58]). We thus were very careful in validating our RNAi approach for knocking down MdBV genes in *M. demolitor* before initiating any functional studies. Our analysis of the ovary transcriptome indicated that all genes of the siRNAi pathway are present in *M. demolitor* and transcribed [7]. Results presented in this study further show that ds-RNA injection into late larval stage *M. demolitor* effectively and specifically knocks down the genes we targeted. While we present the outcome of a number of validation experiments using *vlf-1* as an example, we have conducted similar experiments with other MdBV conserved genes, which all showed the same trends.

With knockdown methods established, we used the baculovirus literature and our proteomic data to select six genes in the MdBV conserved gene set with hypothesized roles in viral transcription (*lef-4, lef-9*), capsid assembly (*vp39, vlf-1*), and envelope formation (*p74, pif-1*). Our rationale for selecting these genes was also driven by the strength of the functional literature for each in baculoviruses, which provided in most cases clear expectations for what a conserved phenotype should be for MdBV. Our results with *lef-4* and *lef-9* strongly support that MdBV produces a baculovirus-like RNA polymerase that preferentially transcribes structural genes. We also note that knockdown of *lef-4* more strongly disabled structural gene expression than knockdown of *lef-9*. This could reflect that as a capping enzyme the effect of knocking down of *lef-4* was further enhanced by degradation of cap-lacking transcripts. The transcription of reporter virus structural genes *vp39* and *p74* was not completely abolished for either *lef-4* or *lef-9* knockdowns. Despite detecting no LEF-9 protein after knockdown on immunoblots, this could reflect incomplete knockdown, and the presence of enough RNA polymerase subunit proteins to produce some functional viral RNA polymerase holoenzyme. Alternatively, viral structural genes may also be transcribed in part by wasp RNA polymerase II. Activity of replication gene transcription compared to relative silence of BV genes that are ultimately packaged into virions suggests that replication genes are transcribed from ancestral viral RNA polymerase promoters whereas virulence genes are not.

The phenotypic effects we observe in response to *vlf-1* knockdown are consistent with this protein being both a structural component and a product required for virion assembly. The defects in morphogenesis of MdBV virions we observe, however, differ somewhat from the defects observed with AcMNPV where knockout of *vlf-1* resulted in formation of tubular structures that appeared to be aberrant capsids that fail to package DNA [40]. The technical approaches to these studies, however, resulted in observations associated with budded virus production and precluded examination of potential defects associated with formation of occlusion-derived virus (see below). In contrast, the severe defects we observed in the assembly of MdBV virions suggest *vlf-1* may be important in both DNA packaging and proper association of capsids with envelopes. Like *vlf-1*, knockdown of *vp39* greatly increased the sensitivity of packaged DNA to DNAse treatment while also reducing infectivity. For both genes, dsRNA treatment did not completely abolish DNAse protection or infectivity of virus particles, which we presume is due to incomplete knockdown of transcript levels. Unlike *vlf-1* though, knockdown of *vp39* did not cause any obvious morphological defects in virion assembly with the exception that virion aggregations in calyx cells were much smaller owing possibly to a reduction in VP39 for production of capsids. Given these observations, we are unclear why knockdown of *vp39* did not reduce virion density in calyx fluid.

Unlike BVs, which produce only one virion type, AcMNPV and most other baculoviruses produce two virion phenotypes named occlusion-derived virus and budded virus [24]. Occlusion-derived virus is embedded in a protein matrix called an occlusion body and is the type that initiates a midgut infection when ingested by a new host. Budded virus in contrast is non-occluded and is the form of the virus that disseminates from the midgut and other cells to systemically infect the insect. Occlusion-derived and budded virus capsids contain the same structural proteins [55] but their envelopes differ greatly with the former assembling *de novo* in host cell nuclei and containing products of several core genes including *per os* infectivity factors. Budded virus in contrast acquires an envelope when budding through host cell plasma membranes, which contains only one or two viral proteins (GP64, F) [55]. Although never occluded, the *de novo* assembly of BVs in calyx cell nuclei together with the envelope components detected in their virions (see above) indicate that BV particles structurally share more features with the occlusion-derived phenotype of baculoviruses. On the other hand, while *per os* infectivity factors are required for infectivity and binding of the occlusion-derived phenotype of AcMNPV to midgut cells, they are not required for infection of cultured cells or host larvae when injected into the hemocoel [49], [59]. We thus were unclear what effect, if any, knockdown of *p74* or *pif-1* might have on infectivity given that BVs infect host insects only when injected into the hemocoel by wasps. Our results reveal no defects in the copy number of MdBV DNA detected in CiE1 cells after infection with a high MOI. Yet, knockdown of each gene resulted in a large decline in the fraction of infected cells that expressed the marker gene GLC1.8. These findings are interesting because they suggest the loss of *per os* infectivity factors from the MdBV envelope results in improper translocation of MdBV to host cell nuclei where transcription of *glc1.8* and other virulence genes occurs. No such activity has previously been associated with *per os* infectivity factors in baculoviruses but intriguingly GP64, the envelope fusion protein of budded virus, has been implicated in affecting baculovirus translocation to host cell nuclei [60].

In addition to targeting six baculovirus-like conserved genes, we also examined the function of nudivirus-like *int-1* because this gene and *vlf-1* are both tyrosine recombinase family members and BV replication requires the excision of proviral genomic DNAs from the wasp genome for packaging into virions. The near complete inhibition of empty locus formation after *vlf-1* and *int-1* knockdown suggests a role for both in proviral DNA excision. At this time we have little understanding of the recombination reactions VLF-1 and INT-1 potentially mediate, although detailed investigation of other tyrosine recombinases suggest they perform recombination events by establishing a synapse between their cognate binding sites and performing two consecutive strand exchanges [61], [62]. If on the same molecule recombination between two binding sites leads to excision of a circular intermediate [63]. Analysis of tyrosine-recombinase structural properties also suggest the mechanism of recombination requires binding of four enzyme monomers, which suggests the possibility for involvement of both *vlf-1* and *int-1* in proviral DNA excision [53]. Additionally, proviral DNA segments that become encapsidated possess direct repeats at the sequence boundaries that abut the flanking wasp DNA, which have been previously hypothesized to contain binding sites for recombinases that mediate excision [18], [19], [30]. Lastly detection of both VLF-1 and INT-1 in virions suggests these factors may also have important functions in parasitized hosts given recent findings that all DNA segments packaged into MdBV virions rapidly integrate into the genome of infected host cells [30].

Taken together, our results provide the first experimental insights into the function of a subset of MdBV genes. We fully recognize that additional experiments will be needed to more fully characterize the function of the individual genes we targeted, but by examining several key genes at once we provide evidence that: 1) RNAi can be used to knock down a number of MdBV genes, and 2) the baculovirus-like genes we targeted exhibit conserved functions despite divergence from nudiviruses more than 100 Mya. At the same time our results with *vlf-1* and *int-1* also identify novel functions unknown from baculoviruses but essential to the biology of BVs. With these results in hand, we are now positioned to undertake both more detailed experiments on these genes as well as studies on nudivirus-like and novel genes in the BV conserved gene set for which expectations about function are less clear.

The arms race between wasps and the hosts wasps parasitize likely underlies the high rates of speciation of BV-carrying braconids [26], [64]. The genetic mechanisms guiding host range evolution in contrast are largely unknown. One hypothesis would be that PDV virion structure has undergone rapid adaptation in response to the different lepidopteran host species each wasp species parasitizes, which could result in high variation in BV virion structure. The similarities thus far found in BV conserved genes together with the functional insights provided here, however, strongly suggest that BV gene functions will be conserved across isolates. Thus, differences in the sequence of BV genes may affect whether a given isolate can infect a given host species but we think it unlikely large differences will be found in the structure of BV virions across isolates. In contrast, the literature already indicates that the virulence genes BVs package into virions vary greatly among isolates associated with phylogenetically distant species of wasps. Thus, we would expect that differences in the virulence genes BVs deliver to hosts strongly affect host range by impacting the ability of wasp offspring to successfully develop. Finally, conservation in the function of the MdBV RNA polymerase and structural proteins suggest that key features in the evolution of BVs into mutualists do not involve radical changes in virion structural products but rather in how: 1) transcription of early factors required for DNA replication and viral transcription are regulated so that replication only occurs in calyx cells, 2) the genome is organized so that only some portions are amplified and packaged into virions, and 3) virulence genes are kept silent in wasps.

## Materials and Methods

### Ethics statement

All studies were approved by the Biological Safety and Animal Care and Use Committee of the University of Georgia and were performed in compliance with relevant institutional policies, National Institutes of Health regulations, Association for the Accreditation of Laboratory Animal care guidelines, and local, state, and federal laws.

### Insects and developmental staging of wasp larvae

*M. demolitor* parasitizes several species of larval stage Lepidoptera including *Chrysodeixis ( = Pseudoplusia) includens*. Both species were reared at 27°C with a 16 h-light:8 h-dark photoperiod. *M. demolitor* has an 11 day developmental period described in detail elsewhere [7]. For this study, *M. demolitor* females were allowed to parasitize *C. includens* larvae, and wasp offspring were then allowed to develop in the host for 6 days. On day 7, wasp larvae emerge from hosts and spin a cocoon within several hours. Cocoons are slightly asymmetrical with the anterior end generally being more elevated and pointed than the posterior end that contains the wasp abdomen. Nine-12 hours after spinning their cocoon, wasps pupate and develop for four more days until emerging as an adult. Adult wasps were then maintained in constant dark at 18°C.

### Proteomic analysis of MdBV virions

Virus was collected from *M. demolitor* ovaries. For the first replicate, 100 whole ovaries were crushed in PBS and the debris was removed by centrifugation. Then, the supernatant containing the virus was spun down at 20,000×g for 5 minutes and washed with PBS 3 times to collect virus particles. For replicate 2, calyx fluid was dissected from 100 dissected ovaries and resuspended in PBS. Virions were then isolated on a sucrose gradient as previously described [31]. Both virus samples were electrophoresed on either a 4–20% or 12.5% Tris-Glycine gel (Lonza). For each sample, the entire lane was cut into four pieces to separate proteins by size. In-gel trypsin digestion was performed for each gel slice, by overnight incubation with trypsin (20 µg/ml) in 20 mM ammonium bicarbonate. Tryptic fragments were extracted with 50% acetonitrile and 0.1% TFA and vacuum dried. Samples (4 µl) were analyzed by an Orbitrap Elite mass spectrometer, coupled to an Easy-nLC II Liquid Chromatography (LC) instrument (Thermo Fisher Scientific). Samples were desalted and pre-concentrated on a C18 Easy LC pre-column (100 um internal diameter (ID) ×2 cm, 5 µm particle packing (PP)). Peptides were eluted from a reverse-phase column (75 um ID ×10 cm, 3 µm PP) with a gradient of 10–35% B for 70 min, 35–95% B for 10 min, 95% B for 5 min (A = 0.1% formic acid in water, B = 0.1% formic acid in acetonitrile) at 300 nl/min. Nanospray ionization was performed with a spray voltage of 2 kV, with a capillary temperature of 200°C. The Orbitrap mass analyzer was used to provide resolutions of 120,000 and 30,000 for MS and MS/MS analyses, respectively. Briefly, a cycle of one full-scan mass spectrum (300–2000 m/z) was performed, followed by continuous cycles of CID and HCD MS/MS spectra acquisitions of the 2 or 5 most abundant peptide ions throughout the LC separation until the candidate ions were exhausted. Data were acquired using Xcalibur software (v2.2, Thermo Fisher Scientific).

Proteins were identified by searching against a custom database “Md” consisting of translated open reading frames (ORFs) greater than 33 amino acids in size from transcripts described in [7] using the Mascot v2.3 algorithm (Matrix Science Inc.). Transcripts can be accessed through NCBI accession numbers JO913492 through JO979916 and JR139425 through JR139430. Data were visualized with ProteomeDiscoverer v1.3 (Thermo Fisher Scientific). Peptides with scores greater than the identity score (*p*<0.05) were considered significant matches. Only ORFs that were matched by at least two peptide spectra were considered positive identifications.

### RNAi assays and quantification of target gene expression

To target individual genes for RNAi knockdown, gene-specific primers were designed with added T7 promoter adaptors to amplify a 300–400 bp template for double-stranded RNA (dsRNA) synthesis (Table S2). cDNA from adult wasp ovaries was amplified using standard PCR and the resulting products were used as template in the MegaScript RNAi Kit (Ambion). Larval stage wasp cocoons were marked within 15 h of spinning and were subsequently injected within 1–3 h. As *M. demolitor* is protandrous with males developing faster than females, the cocoons selected for injections were biased towards later emergence times and thus female wasps. Cocoons were affixed to double-sided tape, and oriented so that the posterior ends were facing in the same direction. Cocoons were pierced with a minuten pin in the abdomen region and wasps were injected through the cocoon with a glass needle directly into the abdomen. Approximately 0.5–1 µl of 2–4 µg/µl dsRNA was injected into each individual. All control wasps were injected with a non-specific dsRNA probe homologous to the bacterial eGFP gene. Wasps were sampled within 24 h of emerging as adults, and ovaries were removed and separated using opthalmic scissors. One ovary half was snap-frozen at −80°C for RNA extraction, and the other was used to assay RNAi phenotypes as described below.

Extraction of total RNA was performed using the QIAGEN RNeasy kit following the standard kit protocol with a 20 min on-column DNAse treatment and elution in 30 µl of RNAse-free H_2_O. RNA concentration was measured using a Nanodrop Spectrophotometer and cDNA was synthesized from a quantity of total RNA normalized across samples. First-strand cDNA synthesis was performed using Invitrogen reagents including the Superscript III enzyme and oligo(dT) primers as outlined by the manufacturer (Invitrogen).

We used quantitative PCR (qPCR) to detect differences in expression of target genes after dsRNA treatment. An absolute standard curve was generated via PCR amplification of the corresponding cDNA for each gene of interest using specific primers (Table S2). Each product was cloned into pSC-A-amp/kan, and after propagating and isolating each plasmid from minipreps, its identity was confirmed by sequencing. Standard curves were generated followed by determination of copy numbers from serially diluted amounts (10^2^ to 10^7^ copies) of each plasmid standard. qPCR was performed on a Rotor-Gene Q using the Rotor-Gene SYBR Green PCR Kit with 1 µM primers and 1 µl of undiluted cDNA per 10 µl reaction (QIAGEN). After 5 minutes of denaturation at 95°C, a two-step amplification cycle with 95°C for 5 sec denaturation and 60°C for 20 sec of annealing and extension was used for 45 cycles. Melting curve analyses were performed to ensure that amplified products were specific for the gene of interest. At least three independently acquired biological replicates were analyzed per stage for each gene, with each sample internally replicated 4 times.

A polyclonal antibody against Lef-9 was generated by PCR amplifying and cloning a portion of MdBV *lef-9* into pET-30-EK-LIC (Novagen) as previously outlined [65]. Briefly, 31% of the *lef-9* coding sequence was amplified and cloned to create an expression product of 22.9 kDa. This construct was confirmed by DNA sequencing, and then expressed in *Escherichia coli* BL21 (DE3) cells grown in 6 L Luria Broth with 50 µg/ml kanamycin at 37°C. The cultures were then induced with 0.025 mM isopropyl-β-d-thiogalactopyranoside (IPTG) for an additional 24 h at 37°C. Bacterial cells were harvested by centrifugation, lysed, and the insoluble recombinant protein was purified from the cell pellet using PerfectPro Ni-NTA (QIAGEN) agarose beads under denaturing conditions. After analysis by SDS–PAGE and immunoblotting using an anti-His monoclonal antibody, the identity of the recombinant protein was validated by mass spectrometry. A polyclonal antibody was then produced by a commercial service (Pacific Immunology) which generated antisera in rabbits by immunizing with ca. 500 µg of affinity purified truncated LEF-9. Antiserum was then purified by nitrocellulose-based immunoaffinity chromatography as previously outlined [65], [66]. The resulting antibody was then used in immunoblotting experiments with control and *lef-9* knockdown wasps by explanting ovaries, separating ovary extracts on 5–20% SDS-PAGE gradient gels and transferring to PVDF (Immobilon). LEF-9 was then visualized using a goat anti-mouse secondary antibody and the ECL Advance kit as previously described [65], [67].

### PCR-based quantification of viral titer, proviral DNA excision, and TEM

The titer of MdBV virions with DNAse-protected episomes of MdBV segment B was quantified by qPCR. One half of an ovary pair for each wasp individual was homogenized with a pestle in 100 µl of DNase buffer from the Roche HighPure RNA Isolation Kit, and NP40 was added to a final concentration of 1% to solubilize wasp cells and virus particle envelopes. After 20 min of gentle rocking, 1 µl of TURBO DNase from the Ambion DNA free kit was added and samples were incubated at 37°C for 40 min to digest all free wasp and episomal viral DNAs. After the addition of EDTA (10 mM) to inactivate the DNase, 250 µg of proteinase K (Roche) and 2% sarcosyl were added to samples, followed by incubation at 62°C for 1 h and by phenol∶chloroform extraction and ethanol precipitation in the presence of 0.3 M sodium acetate, pH 5.2. DNA pellets were resuspended in 30 µl of 10 mM Tris-Cl pH 8.5 and diluted to 1 ng/µl for use as template. Segment B specific primers flanking the point of circularization in the viral segment were used to amplify circularized viral DNA using qPCR as described above and previously [7] (Figure 7B, Table S2). Per-ovary copy numbers were calculated by multiplying the qPCR estimate of copy number for a half ovary by two, and by the dilution factor and elution volume. At least 3 independently acquired biological replicates were performed for each treatment with samples internally replicated 4 times. Quantification of viral segment excision was performed on genomic DNAs extracted from the entire tissue from an ovary half with the proteinase K, sarcosyl, and phenol/chloroform extraction method described above, without a DNAse step. Primers specific for the empty locus for Segment B were used in qPCR to quantify copy number (Figure 7B, Table S2). TEM was performed as in [31].

### Infectivity assays

The CiE1 cell line was maintained as in [43]. The infectivity of virus preparations was measured by counting the number of Segment B viral genome copies in CiE1 cells after 24 hours of incubation [43]. Virus was collected from 24 h old wasps treated dsRNA by puncturing a half ovary in 50 µl of PBS and allowing the virus to dissolve. The entire contents of the droplet were added to a microcentrifuge tube containing 200 ul of Sf900 media with 5% fetal bovine serum and 1% antibiotics. This solution was filtered through a 0.45 µm membrane to remove any bacteria and cellular debris. Fifty µl of each virus preparation, corresponding to 0.1 wasp equivalents or an estimated MOI of 100 for control samples [29] was added to wells in a 24-well cell culture plate containing 10^5^ CiE1 cells per well. Virus particles were incubated with cells for 2 h at room temperature, followed by removal of virus-containing media and the addition of 500 µl of fresh media. CiE1 cells were incubated for an additional 22 hours at 26°C. DNA was isolated from cells following the protocol for quantification of viral titer above without prior DNase treatment. All samples were diluted to a concentration of 50 ng/µl for qPCR amplification of circularized Segment B as described above.

Successful viral gene expression, translation and export of protein products in host cells was quantified by counting the percentage of cells displaying the MdBV protein GLC1.8 on their surface. CiE1 cells were infected with virus preparations as described above, and after 24 hours of total incubation were fixed with 3.7% paraformaldehyde and stained with a murine monoclonal antibody specific for GLC1.8 followed by goat anti-mouse Alexa-fluor 568 secondary antibody (Molecular Probes) as described [44]. One hundred CiE1 cells were counted from a randomly selected field of view using an epifluorescent, phase-contrast microscope (Leica DM IRB).

### Statistical analyses

JMP v10 was used for all statistical analyses. For qPCR analyses, the number of copies of a gene or DNA product were averaged for all technical replicates within a biological replicate. For functional assays, means were calculated from experimental values derived from biological replicates. Each biological replicate represented an individual wasps' ovary. Differences between means of biological replicates were tested using a *t*-test assuming equal variances or ANOVA. Differences between means for experiments with more than two treatments were distinguished using Tukey's HSD test at the *p*<0.05 significance level.

## Supporting Information

Table S1 **BV conserved gene-related proteins detected in MdBV virions.** Conserved gene names and locus identifier in the *M. demolitor* ovary transcriptome are listed to the left [7]. Number of unique peptides from the two proteomic replicates are indicated in the middle columns relative to transcript abundance for each gene that was previously determined as reads per kilobase per million reads mapped (RPKM) in whole adult ovaries [7].
(PDF)

Table S2 **Primers used for dsRNA synthesis template amplification, qPCR assays of expression or segment abundance, and protein expression.**
(PDF)

Figure S1 **Knockdown of** ***vlf-1*** **occurs over a range of ds-** ***vlf-1*** **quantities and is detectable 2 days post-treatment.** (A) *M. demolitor* larvae were injected with ds-*eGFP* (500 ng) or 50 ng, 500 ng or 5 µg of ds-*vlf-1*. The ovaries from individual, newly emerged adults wasps were then dissected and total RNA isolated. The bars in each graph compare copy number of *vlf-1* per ng of total RNA in wasps treated with ds-*eGFP* and each dose of ds-*vlf-1*. (B) Effect of time post-injection of ds-*vlf-1* on transcript knockdown. Larvae were injected with ds-*vlf-1* as described in Fig. 2. Ovaries were then dissected from 2 day old pupae (stage 2), 3 day old pupae (stage 3), 1 day, 3 day and 5 day old adult wasps and total RNA extracted. Level of knockdown is presented as % inhibition relative to ovaries from 1 day adult wasp pretreated with ds-*eGFP*. Error bars, N values, and statistical significance are indicated as defined in Figure 2.
(PDF)

Figure S2 **ds-** ***int-1*** **has no effect on** ***vlf-1*** **while ds-** ***vlf-1-2*** **has a similar knockdown effect as ds-** ***vlf-1*** **.** *M. demolitor* larvae were injected with ds-*eGFP*, ds-*int-1*, or ds-*vlf-1-2* which is specific for the *vlf-1* gene but does not overlap ds-*vlf-1* used in assays shown in Figure 2. The ovaries from individual, newly emerged adults wasps were then dissected and total RNA isolated. (A) The bars in the graph compare copy number of *vlf-1* per ng of total RNA in wasps treated with ds-*eGFP* versus ds-*int-1*. (B) The bars in the graph compare copy number of *vlf-1* per ng of total RNA in wasps treated with ds-*eGFP* versus ds-*vlf-1-2*. (C) Copy number of DNase-protected MdBV episomal genomic segment B in the ovaries of newly emerged *M. demolitor* adult females pretreated with ds-*eGFP* or ds-*vlf-1-2*. Error bars, N values, and statistical significance are indicated as defined in Figure 2.
(PDF)
