# Supplementary material for: Mutualistic Polydnaviruses Share Essential Replication Gene Functions with Pathogenic Ancestors
Source: PLoS Pathog. 2013 May 9;9(5):e1003348. doi: 10.1371/journal.ppat.1003348 (PMC3649998; doi:10.1371/journal.ppat.1003348)
Supplement: Table S1 — BV conserved gene-related proteins detected in MdBV virions. Conserved gene names and locus identifier in the M. demolitor ovary transcriptome are listed to the left [7]. Number of unique peptides from the two proteomic replicates are indicated in the middle columns relative to transcript abundance for each gene that was previously determined as reads per kilobase per million reads mapped (RPKM) in whole adult ovaries [7]. (PDF) [file ppat.1003348.s001.pdf]

Table S1. BV conserved gene-related proteins detected in MdBV virions. Conserved gene names and locus identifier in the *M. demolitor* ovary transcriptome are listed to the left. Number of unique peptides from the two proteomic replicates are indicated in the middle columns relative to transcript abundance for each gene that was previously determined as reads per kilobase per million reads mapped (RPKM).

Gene name	Locus	Number of unique peptides replicate 1	Number of unique peptides replicate 2	Adult ovary mean RPKM
helicase	3891			29
int-1	3978	2	4	53
int-2	1667			48
p47	703			128
lef-4	2025			36
lef-8	*lef-8*			283
lef-9	3218			72
lef-5	539 transcripts 2,7,8,9			159
vlf-1	539 transcripts 1,3,4,5,6,10		2	144
HzNVorf140 (vlf-1b)	3488		2	88
HzNVorf140 (vlf-1b-2)	2965+2966	2	11	358
38K	318	6	18	511
vp91	4098	2	8	210
vp39	*vp39*	19	27	4109
p74	*p74*	13	22	1320
pif-1	*pif-1*	14	17	382
pif-2	226	6	19	448
pif-3	767	2	6	150
19 kDa	1791	7	3	597
odv-e56	13843			5
odv-e56	1447	4	5	329
odv-e56	1083	10	15	217
odv-e66	175	8		405
odv-e66	1139	3	5	91

odv-e66	2331	23	16	197
odv-e66	4186	18	27	207
odv-e66	2730	21	24	244
HzNVorf9-1	*HzNVorf9-1*	10	16	864
HzNVorf9-2	*HzNVorf9-2*	7	14	761
HzNVorf106	332	4	8	508
PmV hypothetical protein	756	4	12	546
ac92	5801	1	4	95
ac92	2756	5		162
HzNVorf64	*HzNVorf64*	3	4	1043
HzNVorf94	318	6	18	511
HzNVorf128	1167			107
17a		2	4	2309
27b	2287	5	11	1823
30b	1266			210
35a	417	20	20	129
35a	420	30	36	249
35a	615 (transcript 4, 9)			277
97a	576	28	30	334
97b	937			309
