# Supplementary material for: Mutualistic Polydnaviruses Share Essential Replication Gene Functions with Pathogenic Ancestors
Source: PLoS Pathog. 2013 May 9;9(5):e1003348. doi: 10.1371/journal.ppat.1003348 (PMC3649998; doi:10.1371/journal.ppat.1003348)
Supplement: Table S2 — Primers used for dsRNA synthesis template amplification, qPCR assays of expression or segment abundance, and protein expression. (PDF) [file ppat.1003348.s002.pdf]

Table S2. Primers used for dsRNA synthesis template amplification, qPCR assays of expression or segment abundance, and protein expression.

Target	Accession numbers	Primer name	Sequence 5'-3'
**dsRNA synthesis primers**			
lef-9	JO947537.1-JO947542.1	Mdlef9RNAiF	TAATACGACTCACTATAGGGGTCGTCCGTATAACATTGGT
		Mdlef9RNAiR	TAATACGACTCACTATAGGGTGGATTTATCAAATCGAAGC
lef-4	JO932223.1-JO932224.1	Mdlef4RNAiF	TAATACGACTCACTATAGGGATGTTCATACCCTTCACCAG
		Mdlef4RNAiR	TAATACGACTCACTATAGGGACGGCAGGGTATGTTTAGTA
vp39	JR139428.1-JR139430.1	vp39_RNAiF	TAATACGACTCACTATAGGGCGGCATCATGCAGTTGTATC
		vp39_RNAiR	TAATACGACTCACTATAGGGATTTGCTGCTTGAGGGTTTG
vlf-1	JO963955.1	vlf1_RNAiF	TAATACGACTCACTATAGGGTTCGGTGTTTCGTCCGTATT
		vlf1_RNAiR	TAATACGACTCACTATAGGGACGGAATTTACGCCACCATA
vlf-1 #2	JO963955.1	vlf-1RNAi2F	TAATACGACTCACTATAGGGAACATGACGGACGCATTAAG
		vlf-1RNAi2R	TAATACGACTCACTATAGGGGAAATCATTGGATACTGATTTTGAA
int-1	JO956053.1	Int_RNAiF	TAATACGACTCACTATAGGGTCTTCCAACACACGATTCCA
		Int_RNAiR	TAATACGACTCACTATAGGGTTTTGGTTCATCAGCAATAAAGC
p74	JO979915.1	Mdp74RNAiF	TAATACGACTCACTATAGGGCCCCACATAATCTCACATCT
		Mdp74RNAiR	TAATACGACTCACTATAGGGTTCTTTTGATCACCGTTCTT
pif-1	JO979916.1	pif-1RNAiF	TAATACGACTCACTATAGGGATTTATCTGCGACGGTGAGG
		pif-1RNAiR	TAATACGACTCACTATAGGGTAATTGCCTTCTTGGGTTGG
eGFP		T7pGEM-Forward	TAATACGACTCACTATAGGGCACGACGTTGTAAAACGAC
		T7pGEM-Reverse	TAATACGACTCACTATAGGGGGATAACAATTTCACACAGG
	**qPCR primers**		
lef-9	JO947537.1-JO947542.1	lef9qPCRF*	CATCTTGATCAGCGTGCAAT
		lef9qPCRR*	ACGTCAGTATTCCCCAGCAC
lef-4	JO932223.1-JO932224.1	lef4qPCRF*	ACCCTTCACCAGGACAACTG
		lef4qPCRR*	AAATAGTACGCGCCACCTTG
vp39	JR139428.1-JR139430.1	vp39qPCRF	CGCTACACGCTATCGATTTG
		vp39qPCRR	CACTGACTGTGCACAAAATTCA
vlf-1	JO963955.1	vlf1_qPCRF	TTTTAAGCCCAAAGCCACAG
		vlf1_qPCRR	AATGGCTTTAAGTCGGGATG
int-1	JO956053.1	IntqPCRF	GCGCAGCAGCTAAGTCATTT
		IntqPCRR	AAAATATCGACGCACGGGTA
p74	JO979915.1	p74F*	TCGGTAATTGATTGGGGAGA
		p74R*	TGCAGCACCAAACAAACAAT
pif-1	JO979916.1	pif-1qPCRF	CTGCAAGTCGTGTCCCTACA
		pif-	ATAACCCAATCGCCGTGATA

		1qPCRR	
Segment B	AY848690.1	SegBLL*	CGTGGATTGACAACGCGTTT
		SegBRR*	TTCTTAGCAGATGATGTCATCGC
		SegBRL*	AGCTTATGTCGACAAGCGCT
		SegBLR2*	TGATTAATTTGTGATACTTCCATGTT
lef-9	JO947537.1- JO947542.1	LEF-9F2	GACGACGACAAGATGTCAATTTTTCAACCACTTTATTTG
		LEF-9R	GAGGAGAAGCCCGGTTTCAATATTCGGACGAATCTAAATCC

*Primer sequences have been previously published in:
Burke GR, Strand MR (2012) Deep sequencing identifies viral and wasp genes with potential roles in replication of *Microplitis demolitor* Bracovirus. J Virol 86: 3293-3306.
