# Supplementary figures and images for: Mutualistic Polydnaviruses Share Essential Replication Gene Functions with Pathogenic Ancestors
Source: PLoS Pathog. 2013 May 9;9(5):e1003348. doi: 10.1371/journal.ppat.1003348 (PMC3649998; doi:10.1371/journal.ppat.1003348)

*vlf-1* copies / ng total RNA

**A**

$F_{3,8}=19.1, p=0.0005$

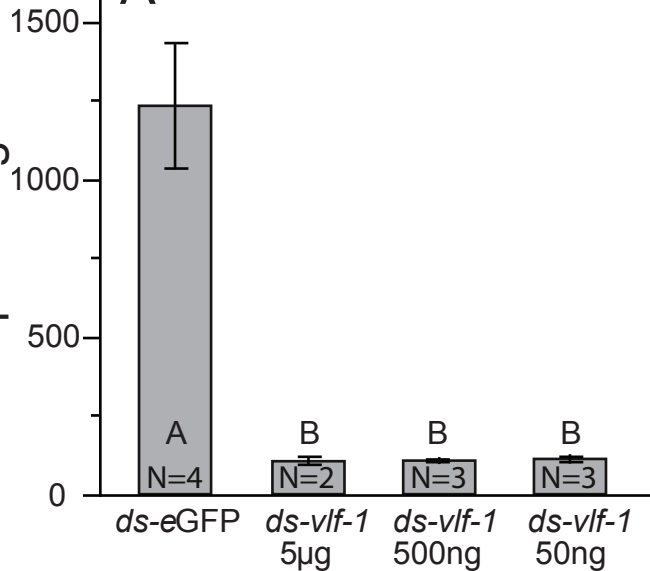

% inhibition

**B**

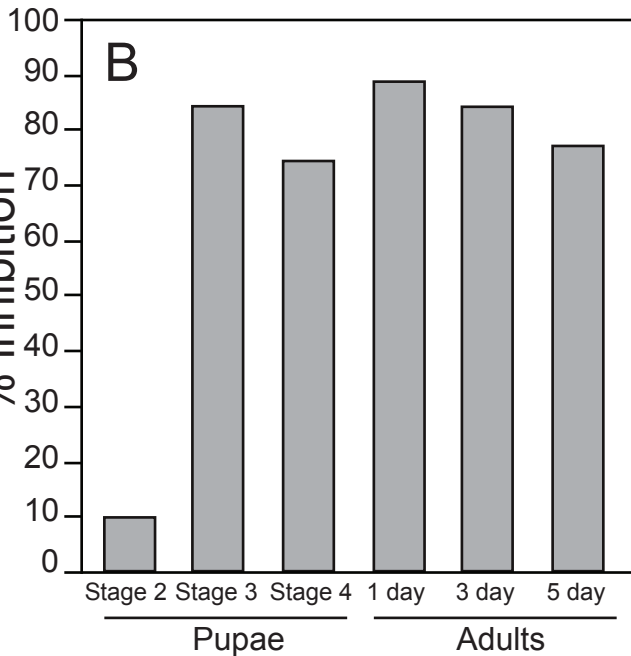

Supplement: Figure S1 — Knockdown of vlf-1 occurs over a range of ds- vlf-1 quantities and is detectable 2 days post-treatment. (A) M. demolitor larvae were injected with ds-eGFP (500 ng) or 50 ng, 500 ng or 5 µg of ds-vlf-1. The ovaries from individual, newly emerged adults wasps were then dissected and total RNA isolated. The bars in each graph compare copy number of vlf-1 per ng of total RNA in wasps treated with ds-eGFP and each dose of ds-vlf-1. (B) Effect of time post-injection of ds-vlf-1 on transcript knockdown. Larvae were injected with ds-vlf-1 as described in Fig. 2. Ovaries were then dissected from 2 day old pupae (stage 2), 3 day old pupae (stage 3), 1 day, 3 day and 5 day old adult wasps and total RNA extracted. Level of knockdown is presented as % inhibition relative to ovaries from 1 day adult wasp pretreated with ds-eGFP. Error bars, N values, and statistical significance are indicated as defined in Figure 2. (PDF) [file ppat.1003348.s003.pdf]

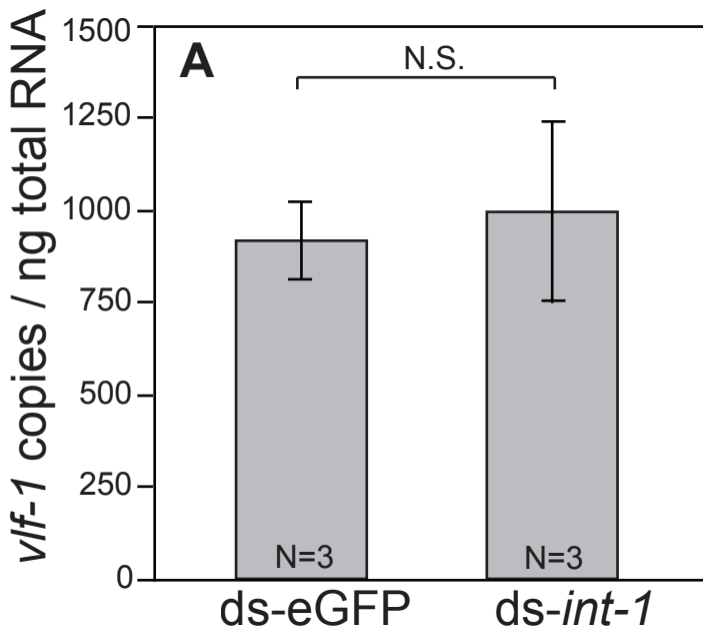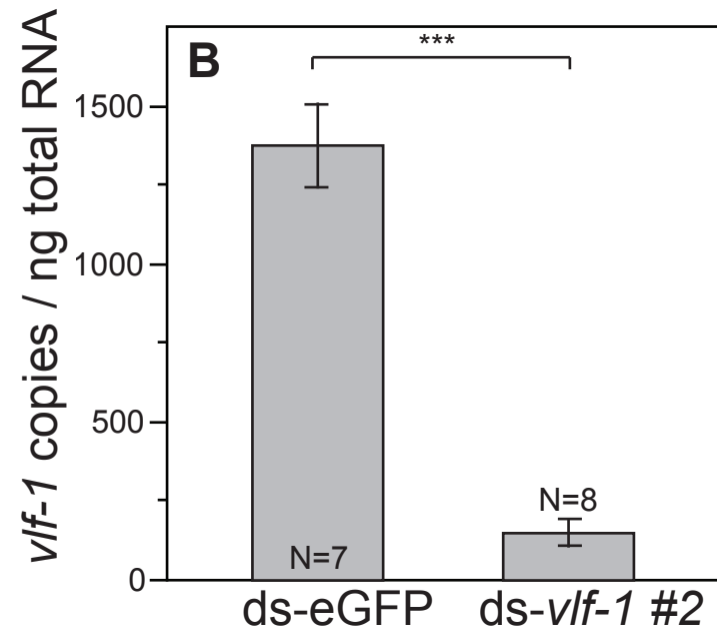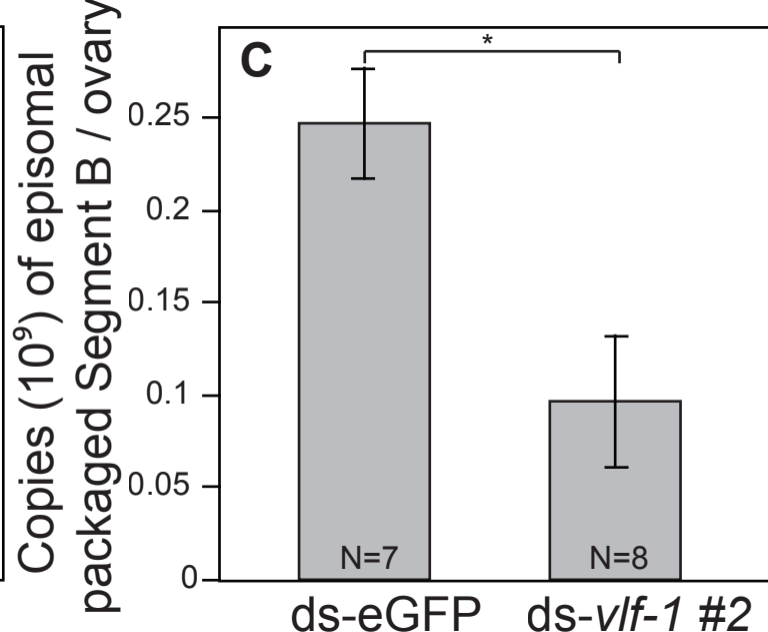

Supplement: Figure S2 — ds- int-1 has no effect on vlf-1 while ds- vlf-1-2 has a similar knockdown effect as ds- vlf-1 . M. demolitor larvae were injected with ds-eGFP, ds-int-1, or ds-vlf-1-2 which is specific for the vlf-1 gene but does not overlap ds-vlf-1 used in assays shown in Figure 2. The ovaries from individual, newly emerged adults wasps were then dissected and total RNA isolated. (A) The bars in the graph compare copy number of vlf-1 per ng of total RNA in wasps treated with ds-eGFP versus ds-int-1. (B) The bars in the graph compare copy number of vlf-1 per ng of total RNA in wasps treated with ds-eGFP versus ds-vlf-1-2. (C) Copy number of DNase-protected MdBV episomal genomic segment B in the ovaries of newly emerged M. demolitor adult females pretreated with ds-eGFP or ds-vlf-1-2. Error bars, N values, and statistical significance are indicated as defined in Figure 2. (PDF) [file ppat.1003348.s004.pdf]
